# Inferior survival outcomes of pancreas transplant alone in uremic patients

**DOI:** 10.1038/s41598-021-00621-y

**Published:** 2021-10-26

**Authors:** Bor-Uei Shyr, Bor-Shiuan Shyr, Shih-Chin Chen, Yi-Ming Shyr, Shin-E. Wang

**Affiliations:** 1 Division of General Surgery, Department of Surgery, Taipei Veterans General Hospital, 10 Fl. 201 Section 2 Shipai Road, Taipei, 112 Taiwan; 2 National Yang Ming Chiao Tung University, Taipei, Taiwan

**Keywords:** Diseases, Endocrinology, Health care, Nephrology

## Abstract

Theoretically, pancreas transplant alone in uremic (PTAU) patients could also be one of the options for those waiting for both pancreas and kidney grafts, but it has never been reported. There were 160 cases of pancreas transplant in this study, including 16% PTAU. The 5-year patient survival was 66.2% after PTAU, 94.5% after SPK, 95.8% after PAK, and 95.4% after PTA. Rejection of pancreas graft was significantly lower in PTAU group (3.8%), followed by 16.7% in pancreas after kidney transplant (PAK), 29.8% in simultaneous pancreas and kidney transplant (SPK) and 37.0% in pancreas transplant alone (PTA). Fasting blood sugar and serum HbA1c levels after PTAU were not significantly different from those by other subgroups. The 5-year death-censored pancreas graft survival was 100% after PTAU and PAK, and 97.0% after SPK and 77.9% after PTA. However, the 5-year death-uncensored pancreas graft survival was 67.0% after PTAU, 100% after PAK, 91.3% after SPK, and 74.0% after PTA. The superior graft survival in the PTAU group was achieved only if deaths with a functioning graft were censored. In conclusion, given the inferior patient survival outcome, PTAU is still not recommended unless SPK and PAK is not available. Although PTAU could be a treatment option for patients with diabetes complicated by end-stage renal disease (ESRD) in terms of surgical risks, endocrine function, and immunological and graft survival outcomes, modification of the organ allocation policies to prioritize SPK transplant in eligible patients should be the prime goal.

## Introduction

Pancreas transplant remains the best option of treatment to achieve long-term physiological euglycemia and insulin independence for patients with labile diabetes mellitus (DM) such as type 1 DM (T1DM). Traditionally, pancreas transplant has been reserved for diabetic patients with end-stage renal disease (ESRD), undergoing simultaneous pancreas-kidney transplant (SPK), and previously receiving a kidney graft and pancreas after kidney transplant (PAK), or those with brittle diabetes without uremia, undergoing pancreas transplant alone (PTA)^1–3^. For patients with labile DM and ESRD, SPK would be a preferred treatment because it can simultaneously provide an insulin-free and dialysis-free life for patients during the same operation^4–9^. Nevertheless, SPK has also been claimed to have the best long-term outcome in diabetic cases with renal failure^10,11^. However, pancreas and kidney transplants are not necessarily to be accomplished at the same time because both pancreas and kidney are not vital organs. Kidney transplant is the well-known treatment of choice for ESRD patients. However, in diabetic patients, the underlying metabolic disturbance will persist and even may get worse after isolated kidney transplant^10^. Currently, PAK has also been an acceptable option for those previously receiving a kidney graft in many centers^1,12,13^. According to the updated Scientific Registry of Transplant Recipients (SRTR) reports^1–3^, majority of the pancreas transplants are for SPK (81% ~ 84%), followed by PTA (9% ~ 11%) and PAK (7% ~ 8%). Theoretically, pancreas transplant alone in uremic patients (PTAU) could also be one of the options for those waiting for both pancreas and kidney grafts. To our knowledge, PTAU has never been reported in the literature.

For diabetic patients with uremia, SPK is undoubtedly the preferred option, and kidney transplant first in PAK is also well documented choice, but how about pancreas transplant first in PTAU? Therefore, the aim of this work was to assess the feasibility and justification of pancreas transplant first in the diabetic patients with uremia, PTAU group, by comparing the surgical risks and outcomes of endocrine, immunology, and pancreas graft survival between PTAU and other traditional transplant subgroups. This work would be the first study regarding the PTAU.

## Methods

Diabetes patients undergoing pancreas transplant from September of 2003 to May of 2020 were included in this study. Perioperative and follow-up data for each patient were prospectively collected and kept in a computer database. This study was approved by our institutional review board, Institutional Review Board (IRB) of Taipei Veterans General Hospital, (IRB-TPEVGH No.: 2020-05-006CC). The study was carried out in accordance with our IRB guidelines and regulations. The informed consent was waived in this retrospective cohort study with anonymity of the data by our institutional review board. The official indications for pancreas transplant in Taiwan include the following: (1) T1DM with diabetic complications such as nephropathy, retinopathy, neuropathy, and cardio-cerebral vasculopathy; (2) T1DM with frequent life-threatening hypoglycemia or hyperglycemia; (3) T1DM with severe disability in school learning, working, and living; and (4) type 2 DM (T2DM) with kidney disease leading to ESRD under insulin control with insulin requirement of less than 1.5 units/kg/day. Based on the patient’s condition, pancreas transplants were classified into 4 subgroups at our institute: PTAU, pancreas transplant alone in uremic patients waiting for both pancreas and kidney transplant; SPK, simultaneous pancreas-kidney transplant; PAK, pancreas after kidney transplant; PTA, pancreas transplant alone. According to the regulations of Taiwan Organ Registry and Sharing Center, the waiting lists for pancreas and kidney grafts are separate, and there are always more than 7,000 uremic patients waiting for a kidney graft, but only about 100 diabetic patients waiting for a pancreas graft (assessed on June 1, 2021 at https://www.torsc.org.tw/about/about_08.jsp). There is always very competition for kidney grafts from deceased donors in our country. A diabetic patient with ESRD might not have pancreas and kidney transplanted at the same time, and some patients who actually needed SPK would even accept pancreas transplant first, so called PTAU, after the procedure is fully explained.

Clinical data and outcomes including early (before discharge) and late (after discharge) complications, surgical mortality, acute and chronic rejections, graft loss, fasting blood sugar, serum hemoglobin A1c (HbA1c), serum C-peptide, and pancreas graft survival were compared between each subgroup of pancreas transplant. In this study, surgical mortality was defined as postoperative death before discharge or within 90 days after operation. Any return to insulin use was counted as pancreas graft failure. Graft loss due to patient death with functioning graft was considered as censor, not event of interest, in graft survival analysis.

The primary endpoint was to compare the patient survivals after the pancreas transplant first in PTAU group with other subgroups. The secondary endpoints were comparisons of pancreas transplant first in PTAU group to other subgroups in terms of endocrine function, immunological, surgical outcomes and graft survival outcomes presenting with and without censoring for death with a functioning graft.

Patients with a positive crossmatch against donor cells were excluded for pancreas transplant. The pancreas grafts were procured in a “‘no touch” technique en bloc with the duodenum. The spleen was routinely separated from the pancreas before aorta cross-clamping. The reason to separate the spleen from pancreas tail before vascular cross-clamping is easier to identify the vessels and to avoid injury to pancreas tail, as compared with the back-table work. We have been doing that procedure from the beginning of our first case, and no detrimental effect is observed so far. Histidine-tryptophan-ketoglutarate solution was used for in situ perfusion, with 4,000 to 6,000 c.c. via aorta and 2,000 to 4,000 c.c. via inferior mesenteric vein. Back-table bench preparation included removal of the peripancreatic fat and arterial Y-graft reconstruction. The graft portal vein was anastomosed end-to-side to the recipient’s distal vena cava with head-up position of the pancreas graft. The superior mesenteric and splenic arteries reconstructed by donor iliac arterial Y-graft at the back-table was anastomosed to the recipient’s right common iliac or external iliac artery. Exocrine drainage was achieved by enteric drainage with a hand-sewn side-to-side duodenojejunostomy 30–50 cm beyond the flexure of Treitz ligament using roux-en-y technique and retroperitoneal placement.

Immunosuppressive treatment was quadruple therapy for all recipients of pancreas transplantation without any specific protocol for each subgroup. Induction therapy included basiliximab (Simulect, Novartis Pharmaceuticals Corp., East Hanover, NJ), 20 mg given on postoperative days 0 to 4, or anti-thymocyte globulin (Thymoglobulin®; Genzyme, Cambridge, Mass., USA) for high risk of rejection such as positive panel-reactive antibody (PRA) and re-transplant, 1 mg/kg daily from postoperative days 1 to 7. Maintenance therapy mainly included administration of tacrolimus (Prograf; Astellas Pharma US, Inc., Deerfield, IL), enteric-coated mycophenolic acid (Myfortic; Novartis Pharmaceuticals Corp., East Hanover, NJ), and prednisolone. Prednisolone was tapered and gradually withdrawn 6 months after transplant. The target trough level for tacrolimus was 8–12 ng/mL during the first year and 6–8 ng/mL thereafter.

Statistical analyses were performed using Statistical Product and Service Solutions (SPSS) version 21.0 software (SPSS Inc., IBM, Armonk, NY, USA). All continuous data were presented as median and mean ± standard deviation (SD), and frequencies were presented when appropriate to the type of data. The mean values of the continuous variables were compared using a two-tailed Student’s t-test. Nonparametric statistical tests were used if the variables did not follow normal distribution. Categorical variables were presented as numbers and percentages. Categorical variables were compared using Pearson’s χ^2^ test or Fisher’s exact test contingency tables. Actuarial graft survival rates were estimated using the Kaplan–Meier method, excluding the primary graft failure and surgical mortality. The log-rank test was used to compare differences in the survival curves. For all analyses, *P* < 0.05 was considered to be statistically significant.

## Results

A total of 160 cases of pancreas transplant were included in this study, including 26 (16%) PTAU, 37 (23%) SPK, 24 PAK (15%), and 73 (46%) PTA. Medical reasons for brain death of deceased donors in pancreas transplants (Table 1). There was no significant difference between PTAU and other subgroups regarding gender, body mass index (BMI), human leukocyte antigen (HLA) mismatch, pretransplant panel reactive antibody (PRA), DM onset age, and dialysis duration, but younger age and shorter DM duration in PTA group as compared with other subgroups with ESRD. Among PTAU group, 13 (50%%) patients were type 2 DM, significantly higher as compared with other subgroups, *P* < 0.001 (Table 2). The median interval between kidney and pancreas transplant in PAK group was 9.5 month, with a range of 1 to 224 months and a mean of 23.6 ± 49.3 months.

**Table 1:** Medical reasons for brain death of deceased donors in pancreas transplants.

Medical reasons for brain death	n	%
**Head injury**		
Traffic accident	66	41.3%
Falling down	23	14.4%
**Hypoxia**		
Choking	6	3.8%
Suicide	18	11.3%
Drowning	2	1.3%
Stroke	36	22.5%
Drug intoxication	5	3.1%
Asthma attack	3	1.9%
Brain tumor	1	0.6%

**Table 2:** Demographics for diabetic patients undergoing pancreas transplant.

	Total	PTAU	SPK	PAK	PTA	*P*-value
Case number	160	26 (16%)	37 (23%)	24 (15%)	73 (46%)	
**Gender**						0.128
Female	90 (56%)	13 (50%)	16 (43%)	13 (54%)	48 (66%)	
**Age, y/o**						< 0.001
Median	33	33	37	38	30	
Range	16–58	25–55	26–58	20–55	16–56	
Mean ± SD	34 ± 9	36 ± 7	38 ± 8	37 ± 9	31 ± 8	
**BMI, kg/m**^**2**^						0.801
Median	21.6	22.0	21.0	22.2	21.0	
Range	12.0–34.1	16.0–30.0	17.1–34.1	16.0–29.0	12.0–31.1	
Mean ± SD	21.9 ± 3.5	22.2 ± 3.0	22.0 ± 4.0	22.3 ± 3.4	21.6 ± 3.4	
Type 2 DM	32 (20%)	13 (50%)	11 (30%)	6 (25%)	2 (3%)	< 0.001
**HLA mismatch**						0.472
Median	3	3	3	3	3	
Range	0–5	0–4	0–5	1–4	0–5	
Mean ± SD	3 ± 1	3 ± 1	3 ± 1	3 ± 1	3 ± 1	
PRA Positive (> 0%)	27 (17%)	6 (23%)	1 (3%)	6 (25%)	14 (19%)	0.060
**DM onset age, year old**						0.424
Median	15	13	17	15	14	
Range	1–43	4–36	8–40	5–28	1–43	
Mean ± SD	16 ± 8	16 ± 7	18 ± 8	15 ± 6	16 ± 8	
**DM duration, year**						< 0.001
Median	18	21	20	22	14	
Range	1–39	7–32	5–39	3–34	1–32	
Mean ± SD	17 ± 8	20 ± 5	19 ± 7	22 ± 8	14 ± 7	
**Dialysis duration, month**						0.181
Median	18.0	19.0	15.0	29.0	N/A	
Range	0.8–168.0	2.0–60.0	0.8–72.0	3.0–168.0		
Mean ± SD	25.5 ± 26.6	25.5 ± 26.6	23.3 ± 23.7	36.8 ± 42.9		

*PTAU* pancreas transplant alone in uremic patients, *SPK* simultaneous pancreas and kidney transplant, *PTA* pancreas transplant alone, *PAK* pancreas after kidney transplant, *BMI* body mass index, *HLA* human leukocyte antigen, *PRA* panel reactive antibody, *DM* diabetes mellitus.

**Figure 1:**
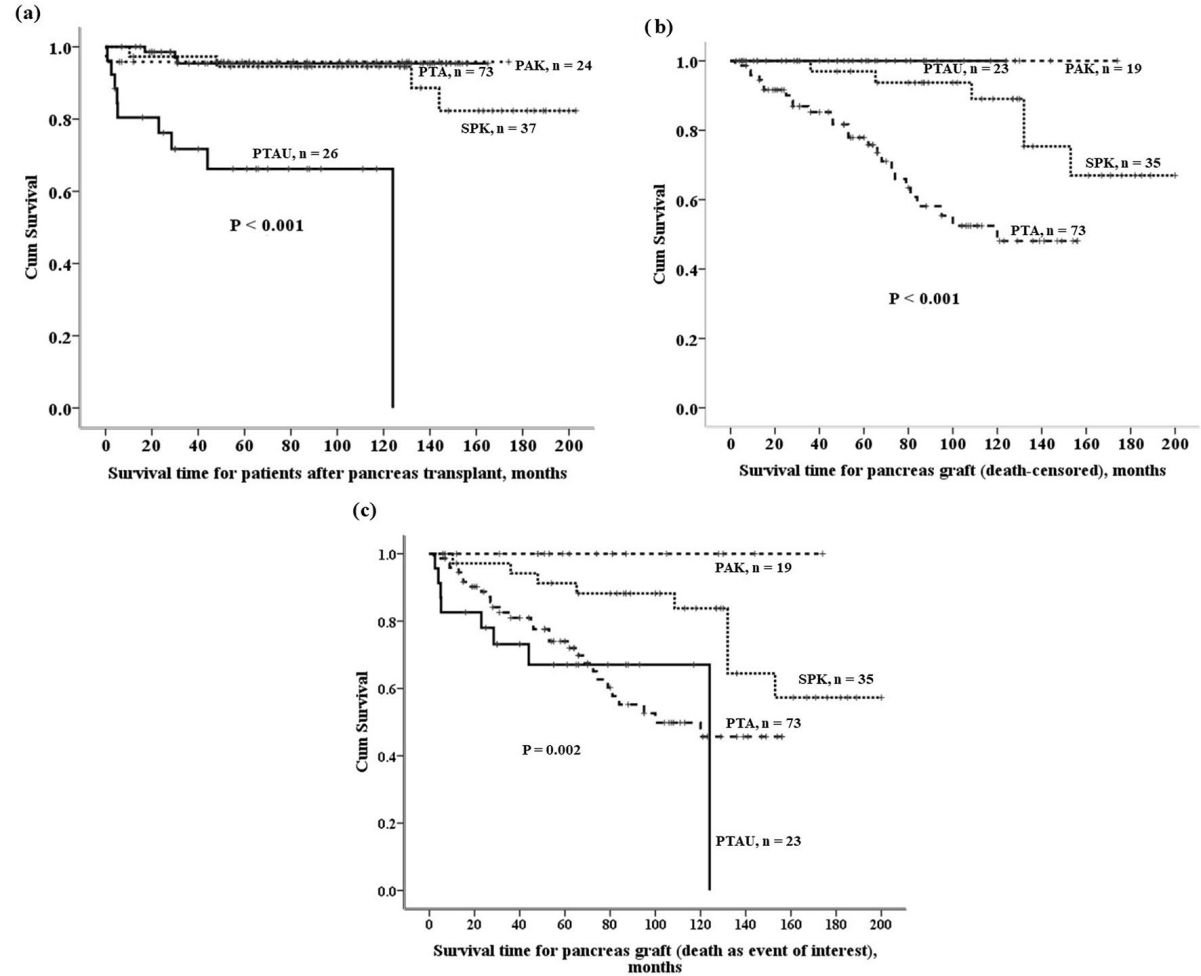
Patient and graft survival curves after pancreas transplant. (**a**) Patient survivals after pancreas transplants. (**b**) Pancreas graft survivals (death-censored) after pancreas transplants. Graft loss due to patient death with functioning graft was considered as censor, not event of interest. (**c**) Pancreas graft survivals (death-uncensored) after pancreas transplants. Graft loss due to patient death with functioning graft was considered as event of interest. The superior graft survival in the PTAU group was achieved only if deaths with a functioning graft were censored.

The 5-year patient survival was 66.2% after PTAU, 94.5% after SPK, 95.8% after PAK, and 95.4% after PTA (Table 3). Patient survival outcome after PTAU was worse than other pancreas transplant subgroups (Fig. 1a). The causes of patient death in PTAU group included 3 cerebrovascular accident, 2 acute myocardial infarction, 2 sepsis, 1 hepatic failure due to hepatitis and 1 unknown cause.

**Table 3:** Survivals for patients after pancreas transplant.

	Total	PTAU	SPK	PAK	PTA	*P*-value
Case number*	160	26	37	24	73	< 0.001
Median, month	87	42	129	68	86	
Range, month	0.3–203	1–124	10–203	0.3–174	7–165	
Mean ± SD, month	88.1 ± 53.0	49.0 ± 38.5	125.2 ± 51.0	74.2 ± 47.3	87.7 ± 48.7	
1-year survival	95.6%	80.4%	97.3%	95.8%	100%	
5-year survival	90.6%	66.2%	94.5%	95.8%	95.4%	
10-year survival	88.8%	66.2%	94.5%	95.8%	95.4%	

*PTAU* pancreas transplant alone in uremic patients, *SPK* simultaneous pancreas and kidney transplant, *PTA* pancreas transplant alone, *PAK* pancreas after kidney transplant, *Surgical mortality was included.

Pancreas graft survival outcome after PTA was worse than other pancreas transplant subgroups if graft loss due to patient death with functioning graft was considered as censor, not event of interest, *P* < 0.001 (Fig. 1b), whereas pancreas graft survival outcome after PTAU was worse than other pancreas transplant subgroups if graft loss due to patient death with functioning graft was considered as event of interest, *P* = 0.002 (Fig. 1c). The 5-year death-censored pancreas graft survival was 100% after PTAU and PAK, and 97.0% after SPK and 77.9% after PTA. However, the 5-year death-uncensored pancreas graft survival was 67.0% after PTAU, 100% after PAK, 91.3% after SPK, and 74.0% after PTA (Table 4). The superior graft survival in the PTAU group was achieved only if deaths with a functioning graft were censored.

**Table 4:** Survival of pancreas grafts after pancreas transplant.

	Total	PTAU	SPK	PAK	PTA	*P*-value
Death-censored graft survival*						< 0.001
Case number	150	23	35	19	73	
Median, month	66	44	118	62	62	
Range, month	2–200	3–124	10–200	6–174	2—156	
Mean ± SD, month	75.9 ± 49.0	50.4 ± 36.2	113.5 ± 49.7	72.7 ± 46.9	66.8 ± 43.4	
1-year survival	97.9%	100%	100%	100%	95.9%	
5-year survival	88.2%	100%	97.0%	100%	77.9%	
10-year survival	70.6%	N/A	89.1%	100%	48.1%	
**Death-uncensored graft survival**						0.002
1-year survival	94.6%	82.6%	97.1%	100%	95.9%	
5-year survival	80.3%	67.0%	91.3%	100%	74.0%	
10-year survival	64.2%	67.0%	83.8%	100%	45.70%	

*PTAU* pancreas transplant alone in uremic patients, *SPK* simultaneous pancreas and kidney transplant, *PTA* pancreas transplant alone, *PAK* pancreas after kidney transplant, *N/A* not available, *technique failure was not included, and graft loss due to patient death with functioning graft was considered as censor.

The surgical and immunological outcomes were listed in Table 5. There was no significant difference in surgical outcomes between PTAU and other subgroups including cold ischemic time, warm ischemic time, overall complications, surgical mortality, hospital stay and cost. The early complication was lower in PTA group as compared with other subgroups with ESRD. Rejection of pancreas graft was much lower, 3.8% in PTAU group, followed by 16.7% in PAK, 29.8% in SPK and 37.0% in PTA, *P* = 0.006. There was no chronic rejection in PTAU and PAK groups, *P* = 0.045. Pancreas graft loss rate was higher (38.5%) in PTAU and PTA groups, *P* < 0.001. Majority (9/10) of pancreas graft loss in PTAU group was due to patient death with functioning graft (34.6%) and the remaining one (1/10) was resulted from technical failure with graft hemorrhage (3.8%) after revascularization of graft during operation. Majority (16/28) of pancreas graft loss in PTA group was chronic rejection (21.9%).

**Table 5:** Surgical and immunological outcomes for diabetic patients after pancreas transplant.

	Total	PTAU	SPK	PAK	PTA	*P*-value
Case number	160	26 (16%)	37 (23%)	24 (15%)	73 (46%)	
**Cold ischemic time, min**						0.483
Median	370	367	450	355	360	
Range	102–995	192–624	228–767	163–995	102–736	
Mean ± SD	399 ± 150	368 ± 127	466 ± 147	379 ± 172	382 ± 145	
**Warm ischemic time, min**						0.412
Median	38	38	39	37	37	
Range	23–86	25–49	27–60	27–67	23–86	
Mean ± SD	39 ± 10	37 ± 6	41 ± 8	40 ± 11	39 ± 11	
**Complications**	112 (70.0%)	18 (69.2%)	30 (81.1%)	17 (70.8%)	47 (64.4%)	0.351
Early complication	74 (46.3%)	13 (50.0%)	23 (62.2%)	15 (62.5%)	23 (31.5%)	0.005
Late complication	79 (49.4%)	10 (38.5%)	21 (56.8%)	10 (41.7%)	38 (52.1.0%)	0.419
Surgical mortality	4 (2.5%)	1 (3.8%)	1 (2.7%)	2 (8.3%)	0	0.143
**Rejection**	41 (25.6%)	1 (3.8%)	9 (24.3%)	4 (16.7%)	27 (37.0%)	0.006
Acute rejection	30 (18.8%)	1 (3.8%)	6 (16.2%)	4 (16.7%)	19 (26.0%)	0.088
Chronic rejection	15 (9.4%)	0	4 (10.8%)	0	11 (15.1%)	0.045
**Graft loss**	54 (33.8%)	10 (38.5%)	11 (29.8%)	5 (20.8%)	28 (38.4%)	< 0.001
Acute rejection	7 (4.4%)	0	1 (2.7%)	0	6 (8.2%)	
Chronic rejection	21 (13.1%)	0	5 (13.5%)	0	16 (21.9%)	
Death with functioning graft	18 (11.3%)	9 (34.6%)	4 (10.8%)	2 (8.3%)	3 (4.1%)	
Graft hemorrhage	3 (1.9%)	1 (3.8%)	0	2 (8.3%)	0	
Primary nonfunction	3 (1.9%)	0	1 (2.7%)	1 (4.2%)	1 (1.4%)	
Unknown	2 (1.3%)	0	0	0	2 (2.7%)	
**Hospital stay, day**						0.423
Median	15	16	17	19	12	
Range	7–112	9–78	7–112	8–60	8–68	
Mean ± SD	19 ± 14	23 ± 16	21 ± 17	23 ± 13	16 ± 11	
**Hospital cost, USD**						0.471
Median	25,024	27,608	26,952	28,793	22,747	
Range	13,926–63,623	21,862–48,342	15,625–60,466	17,533–51,229	13,926–63,623	
Mean ± SD	26,775 ± 8,729	30,191 ± 7,839	28,745 ± 9,102	28,958 ± 9,102	24,083 ± 7,572	

*PTAU* pancreas transplant alone in uremic patients, *SPK* simultaneous pancreas and kidney transplant, *PTA* pancreas transplant alone, *PAK* pancreas after kidney transplant, *USD* United States Dollar.

**Figure 2:**
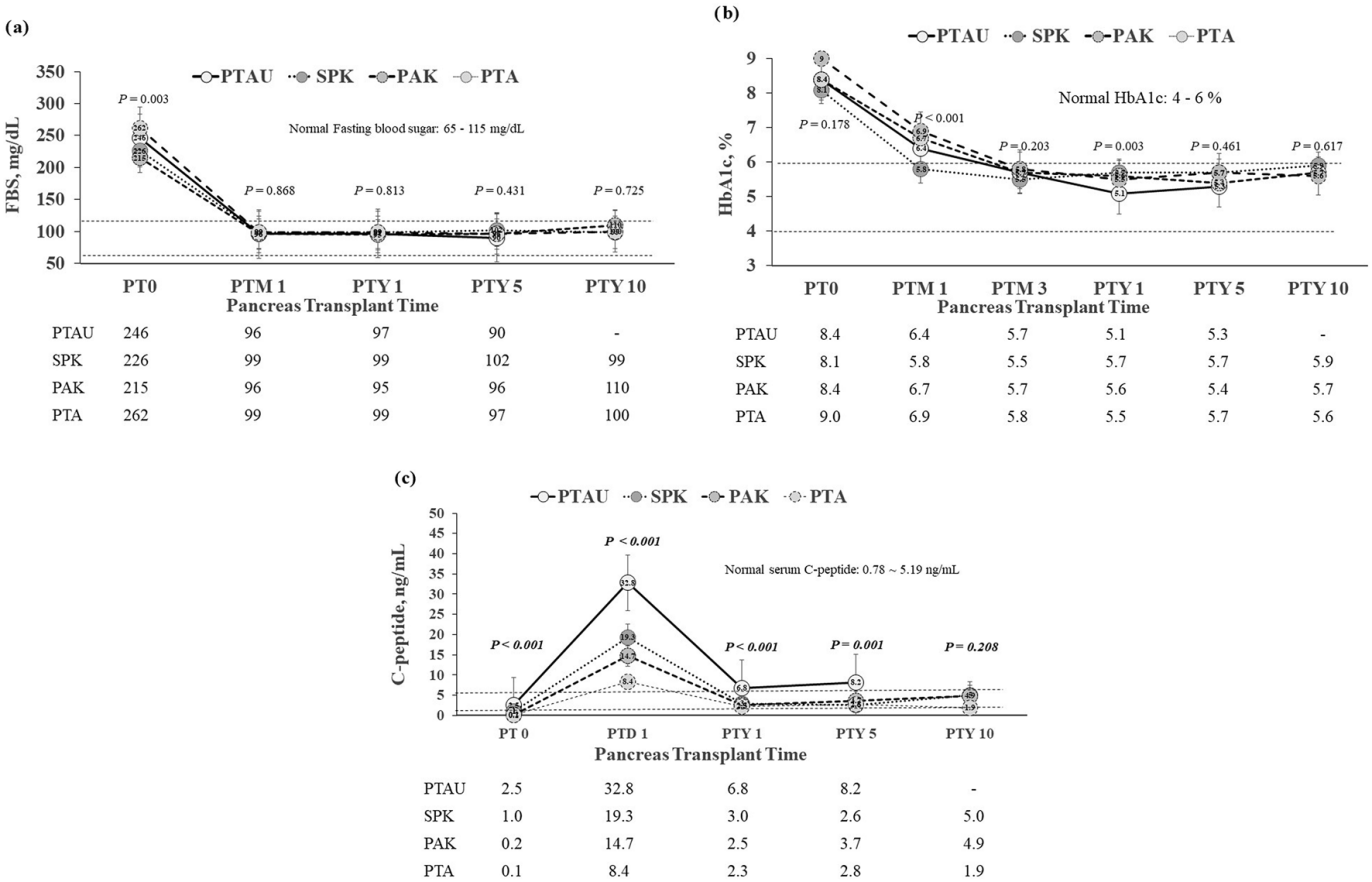
Endocrine function after pancreas transplants. (**a**) Fasting blood sugar (FBS) levels after pancreas transplants. (**b**) Hemoglobin A1c (HbA1c) levels after pancreas transplants. (**c**) Serum C-peptide levels after pancreas transplants. *PT 0* post-transplant day 0, *PTD* post-transplant day, *PTM* post-transplant month, *PTY* post-transplant year.

The short-term and long-term fasting blood sugar (FBS) values after PTAU were not significantly different from those by other pancreas transplant subgroups (Fig. 2a). There was also no significant difference regarding the serum hemoglobin A1c (HbA1c) levels between each subgroup at each post-transplant follow-up time, except that at 1 year follow-up after PTAU, with a median HbA1c of 5.1%, significantly lower than other subgroups, *P* = 0.003 (Fig. 2b). The serum C-peptide levels were significantly higher in PTAU group than those by other pancreas transplant subgroups at each post-transplant follow-up time (Fig. 2c).

## Discussion

Pancreas transplant remains the most effective option of treatment to achieve and maintain physiological euglycemia, and, moreover, to halt or even potentially reverse the secondary complications related to diabetes. Significant improvements in quality of life and better life expectancy make it in the longer term, a lifesaving procedure compared to waiting candidates^14^. For diabetic patients with ESRD, SPK has been a standard treatment to provide a healthier lifestyle without the burden of dialysis and insulin therapy^4,10^. Pancreas graft allocation system may be different from country to country^1,4,10,15^. The new pancreas allocation system went into effect on October 30, 2014 in the America, run by Organ Procurement and Transplantation Network (OPTN)/United Network for Organ Sharing (UNOS)^15^. All solid‐organ pancreas transplant types (SPK, PTA, and PAK) were combined into a single waiting list^15^. According to OPTN/SRTR 2018 Annual Data Report, 84% of pancreas transplants were SPK, 9% PTA, and 7% PAK^1^. No PTAU has been reported in the literature to date. In Taiwan, the pancreas and kidney allocation systems are separate and different. Pancreas and kidney waiting lists are not combined together, and the diabetic candidates (about 100 patients) on the pancreas waiting list are always much outnumbered by uremic candidates (> 7,000 patients) on the kidney waiting list (assessed on June 1, 2021 at https://www.torsc.org.tw/about/about_08.jsp). Therefore, there is very competition for a kidney graft from a deceased donor in Taiwan; a diabetic patient with ESRD might not have pancreas and kidney transplanted at the same time, and some patients who actually needed SPK would even undergo PTAU. These PTAU patients after pancreas transplant first in diabetic patient with uremia are still on the regular kidney transplant waiting list according to Taiwan organ allocation system. Nevertheless, the priority of pancreas transplant is SPK, PAK, PTA and PTAU. Therefore, when there is a suitable pancreas graft without an available kidney graft from the same donor, the pancreas graft might go to the patients on the PTA group. That is why the proportion of PTA is so high in our series.

However, patient survival outcome in PTAU group is inferior to other pancreas transplant subgroups. PTAU patients with unresolving uremic condition seem to be more vulnerable to cardiac events, cerebrovascular accidents, infections and malignancies, like seen in our series. These risks could be attributed to the underlying uremia-associated immune deficiency as mentioned earlier^16–20^.

Technically, PTAU could be performed with similar operation times (cold and warm ischemic times) and without increasing surgical risks in terms of surgical morbidity and mortality, as compared with other pancreas transplant subgroups. Meanwhile, PTAU did not significantly increase the hospital stay and cost. According to OPTN/SRTR 2018 Annual Data Report^1^, incidence of a first rejection episode 1 year after pancreas transplant was 12.4% for SPK, 19.2% for PTA and 11.7% for PAK respectively. The higher incidence of rejection after PTA might reflect a trend toward protocol biopsies based on historically higher incidence of rejection and lack of reliable markers for rejection in the absence of a simultaneously transplanted kidney^1^. This study also showed higher incidence of both acute (26.0%) and chronic (15.1%) rejection in PTA group; however, only 1 case of acute rejection (3.8%) and 0% chronic rejection occurred in PTAU. Although both PTAU and PTA were eventually associated with higher graft loss rate, 38.5% and 38.4% respectively, the causes of graft loss for these two groups were different. Most of graft loss in PTA group resulted from rejection (30.1%), including chronic (21.9%) and acute (8.2%) rejection, whereas the leading cause of graft loss in PTAU group was patient death with functioning graft (34.6%). It has been claimed that uremia-associated immune deficiency is a well-known complication of loss of renal function and contributes significantly to the overall mortality and morbidity of patients with ESRD^16,17^. Immunologically, ESRD is associated with functional defects in virtually all cell populations of both the innate and adaptive immune systems. The ESRD-related changes in the immune system resemble immunological aging in the very old healthy individuals, a concept known as premature immunological aging^16–18^. Chronic inflammation and increased oxidative stress probably contribute to the underlying the uremia-associated immune deficiency. Although these disorders are complex, yet thoroughly unknown, the uremia-associated immune deficiency would result in a decreased vaccination response, cardiovascular events, more infections and increased susceptibility for malignancies^16,17,19,20^. Therefore, the low graft rejection in PTAU patients might be a reflection of the underlying uremia-associated immune deficiency/inertia. In the other hand, the uremia-associated immune deficiency might play a substantial role in the clinical implications, and could be the main contributing factor for the higher rate of patient death with functioning graft after a successful PTAU in this study.

Endocrine function of the pancreas graft would be the prime concern after pancreas transplant. PTAU could also effectively provide similar endocrine function in terms of fasting blood sugar and HbA1c levels, compared to other pancreas transplant subgroups in this study. Nevertheless, the serum c-peptide levels in PTAU group are even significantly higher than those in other pancreas transplant subgroups. This finding of higher serum c-peptide levels could a reflection of more T2DM patients (50%) in PTAU groups because T2DM patients tend to be associated with hyperinsulinemia ^21^. One of the reasons for superior levels of c-peptide in PTAU group is probably biased by persistent uremia, which is known to increase c-peptide level.

Pancreas graft survival outcome after PTAU is essentially similar to SPK and PAK, and superior to PTA by our study. These findings imply that the uremia-associated immune deficiency/inertia in PTAU, SPK and PAK patients might play a role in the lower incidence of rejection, which contribute to the better graft survival outcome, as compared with PTA group. It seems that the uremia-associated immune deficiency/inertia in PTAU patients would trade a superior pancreas graft survival for an inferior patient survival outcome. Therefore, subsequent kidney transplant following a successful pancreas transplant should be encouraged whenever possible.

Limitation of this study is no data of kidney grafts to be presented in PTAU group. It has been very competitive for our uremic patients to have a kidney graft for transplant because the pancreas and kidney waiting lists are separate according to our organ allocation policy. Actually, no one in PTAU group undergo subsequent kidney transplant so far during the follow-up period although they had been kept waiting for that. Therefore, the term “pancreas before kidney (PBK)” transplant would be some kind of misnomer without subsequent kidney transplant. Probably, pancreas transplant alone in uremic patients (PTAU) could be more clearly understandable.

## Conclusion

PTAU could be a treatment option for patients with diabetes complicated by ESRD in terms of surgical risks, endocrine function, and immunological and graft survival outcomes. However, given the inferior patient survival outcome, pancreas transplant alone in uremic patients in PTAU group is still not recommended whenever possible, unless SPK and PAK is not available due to constraints from the kidney allocation policies. Therefore, the rationale for PTAU would be amelioration of short-term diabetic morbidity to allow for patients to remain medically stable to wait for a kidney transplant and also to further avoid the long-term complications associated with DM, and also seen in SPK and PAK patients. Subsequent kidney transplant following successful pancreas transplant should be encouraged whenever possible because PTAU patients might be more vulnerable to cardiac events, cerebrovascular accident, infection, and malignancy. Nevertheless, modification of the organ allocation policies to prioritize SPK transplant in eligible patients should be the prime goal and PTAU should be avoided.

## Data Availability

The data that support the findings of this study are available on request from the corresponding author. The data are not publicly available due to privacy or ethical restrictions.

## Abbreviations

DM: Diabetes mellitus
T1DM: Type 1 DM
SPK: Simultaneous pancreas-kidney transplant
PAK: Kidney graft and pancreas after kidney transplant
PTA: Pancreas transplant alone
PTAU: Pancreas transplant alone in uremic patients
BMI: Body mass index
HLA: Human leukocyte antigen
C-peptide: Connecting-peptide
SRTR: Scientific Registry of Transplant Recipients
ESRD: End-stage renal disease
HbA1c: Hemoglobin A1c
PRA: Panel reactive antibody
